# Potential Impact of Umbilical-Cord-Blood Procalcitonin-Based Algorithm on Antibiotics Exposure in Neonates With Suspected Early-Onset Sepsis

**DOI:** 10.3389/fped.2020.00127

**Published:** 2020-04-17

**Authors:** Noémie Huetz, Elise Launay, Géraldine Gascoin, Bertrand Leboucher, Christophe Savagner, Jean B. Muller, Sophie Denizot, Cécile Boscher, Jocelyne Caillon, Damien Masson, Christèle Gras Le Guen

**Affiliations:** ^1^Department of Neonatal Medicine, Angers University Hospital, Angers, France; ^2^Clinical Investigation Center 004, INSERM 1413, Nantes University Hospital, Nantes, France; ^3^Loire Infant Follow-Up Team (LIFT) Network, Pays de Loire, France; ^4^Department of Neonatal Medicine, Nantes University Hospital, Nantes, France; ^5^Department of Neonatology, Polyclinique de l'Atlantique, St Herblain, France; ^6^Laboratory of Microbiology, Nantes University Hospital, Nantes, France; ^7^Laboratory of Biochemistry, Nantes University Hospital, Nantes, France

**Keywords:** early-onset neonatal infection, risk stratification, screening tool, decrease antibiotics exposure, biomarkers, newborns, antibiotic stewardship

## Abstract

**Context:** The incidence of early-onset neonatal infection has greatly decreased, but a new diagnostic approach is needed to avoid overdiagnosis and overtreatment. The aim of this study was to assess the potential impact of an algorithm incorporating umbilical-cord-blood procalcitonin (PCT) level on neonatal antibiotics prescription rate as compared with current practice.

**Material and methods:** We conducted a prospective study in three maternity wards in France. All term and preterm neonates with the usual risk factors for neonatal group B *Streptococcus* infection were eligible for umbilical-cord-blood PCT testing. We compared the proportion of neonates who were exposed early to antibiotics (before 6 days of life) to that of neonates for whom antibiotics prescription would be indicated according to the PCT-based algorithm.

**Results:** Among the 3,080 neonates included, 1 neonate presented with certain infection and 38 neonates with probable infection. The global antibiotics prescription rate was 4.6% [95% confidence interval (CI), 4.1–5]. With the PCT-based algorithm, the potential decrease in prescription rate would be 1.8% (95% CI, 1.3–2.3), corresponding to a 39% (95% CI, 37.3–40.7) relative reduction in antibiotics exposure (*p* < 0.05).

**Conclusion:** These results suggest that the umbilical-cord-blood PCT-based algorithm could significantly help the clinicians in their antibiotic prescription decision to decrease neonatal antibiotics exposure as compared with current practice. If validated in a larger interventional randomized study, this approach could help clinicians stratify the risk of early-onset neonatal infection and initiate early antibiotics treatment in newborns at high risk of infection while limiting the deleterious effects of useless prescriptions in non-infected newborns.

## Introduction

Early-onset neonatal infection (EONI) remains one of the leading causes of neonatal morbidity and mortality (1) because of the immune weakness of newborns (2) and fast evolution of sepsis. This pathology is considered a diagnostic and therapeutic emergency. However, although EONI is often suspected, it is diagnosed in only 0.8 to 1/1,000 births (3). Because of the non-specificity of clinical signs of EONI, pediatricians often empirically start antibiotics treatment in well-newborns with suspected EONI but who present none or only a few clinical symptoms before receiving results of bacteriological culture and inflammatory markers. This situation leads to a considerable number of newborns unnecessarily exposed to antibiotics. Stocker et al. reported empiric antibiotics treatment administered for ≥ 72 h to 82% of a cohort of neonates with suspected EONI, but only 18% of cases were finally classified as probable infections and 1% as proven infections (4). A French study reported antibiotics treatment instituted during the first 48 h of life in 3.9% of 1,217 newborns: 88% were not infected, 11% were colonized, 1% presented probable infection, and none presented certain infection (5). Such therapeutic strategies have consequences and are responsible for perturbations in the newborn microbiota with possible long-term consequences such as autoimmune, allergic, or metabolic pathologies (6, 7); resistant bacteria (8); nosocomial infection and necrotizing enterocolitis (9); immune system maturation; mother/newborn separation; and increased cost (6, 10, 11).

The diagnostic value of procalcitonin (PCT) level has been reported in adult bacterial infection (12) and in children (13). PCT level also seems a reliable marker for bacterial infection in full-term (14) and preterm (15) newborns. However, studies by Chiesa et al. (16) and Turner et al. (17) point to the difficulty of interpreting this marker in the early neonatal period because of the physiological increase in level during the first 48–72 h of life, despite this being the precise period when the diagnosis of EONI should be established. In a study of 2,151 newborns with suspected EONI (15), the PCT value assessed by umbilical cord blood level preceded this physiological peak, and a cutoff level of 0.6 ng/ml could distinguish infected and healthy newborns. A recent retrospective and monocentric study proposed a new PCT-based algorithm to stratify the EONI risk and suggested a potentially significant reduction in antibiotics prescription rate with the algorithm without missing infection diagnoses (7). In this previous study, the PCT-based algorithm had a 90% (95% CI, 76.9–100) sensibility and a 91.7% (95% CI, 90.6–92.8).

The aim of this study was to assess the potential impact of this new PCT-based algorithm on neonatal antibiotics prescriptions as compared with current practice in a prospective and multicentric population of neonates with suspected EONI.

## Materials and Methods

We conducted a multicentric, prospective, and non-interventional study to analyze umbilical-cord-blood PCT in three hospital birth centers. This was an ancillary study of the HEMOCORD study that assessed the concordance between umbilical-cord-blood and venous blood culture in EONI. Parental informed consent was obtained for each newborn. The study was approved by the local clinical research committee of Nantes University Hospital.

### Inclusion and Exclusion Criteria

All term and preterm newborns > 36 weeks' gestation with suspected EONI who were born during the study period were eligible. The risk factors of suspected EONI were those proposed by international and French national consensus guidelines (18): clinical suspicion of chorioamnionitis (fever, painful uterine contraction), intrapartum maternal fever > 38°C, infected twin, spontaneous premature delivery at <37 weeks' gestation, prolonged rupture of membrane > 12 h, maternal colonization with group B *Streptococcus* (GBS) without full prophylactic antibiotics treatment, and signs of fetal asphyxia (anomalies of fetal heart rhythm, meconium-stained amniotic fluid). We secondarily excluded newborns without available umbilical-cord-blood PCT level (missed sampling or technical impossibility) or without parental consent.

### Newborn Classification

Three experts (two neonatologists not involved in the care of newborns and one infectiology specialist), who were unaware of the PCT value or the diagnosis and treatment performed in clinical practice, *a posteriori* defined two groups of newborns according to clinical, biological, and bacterial criteria [based on national and international consensus guidelines (19)]: (1) infected newborns were defined by a positive central (blood or cerebrospinal fluid) microbiological sample (proven infection) or by the association of postnatal biological signs of sepsis and clinical signs of infection or gastric fluid positive for a pathogen germ (probable infection), and (2) non-infected newborns were defined by no clinical or postnatal biological signs of sepsis; gastric fluid was negative or was positive but with non-pathogen germs.

The clinical signs of sepsis were those proposed by international and French consensus guidelines (1, 20): fever (>37.8°C) or hypothermia (<35°C), tachycardia or bradycardia, arterial hypotension, poor perfusion, respiratory distress, apnea, seizure, floppy infant, bulging fontanel, irritability, lethargy, and purpura. The biological signs of sepsis were defined by the following criteria, according to consensus guidelines: white blood cell count > 25.10^9^/L or <4.10^9^/L, blood platelet count <150.10^9^/L, immature neutrophil proportion > 5%, and C-reactive protein (CRP) level ≥ 20 mg/L.

The PCT value was known by clinicians only in Center C, but the experts were blinded to the result when they classified the newborns according to available guidelines.

### Algorithms

**Figure 1:**
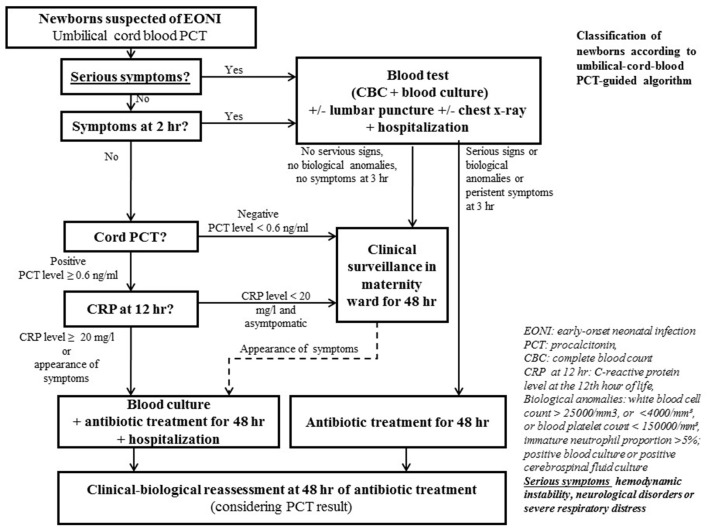
Procalcitonin (PCT)-based algorithm (2017).

The usual French national guidelines in effect during this study recommended an approach to evaluate the risk of neonatal infection based on risk factors and clinical and biological values (1); new French health authority guidelines recently published (2017) proposed an approach based on only risk factors and clinical values for newborns > 34 weeks' gestation with suspected EONI (21). The alternative algorithm proposed by S. Lencot and evaluated in this study included supplementary umbilical-cord-blood PCT and CRP levels to identify newborns with suspected EONI (7) (Figure 1). The PCT value was considered positive with PCT level ≥ 0.6 ng/ml and the CRP value positive with CRP level ≥ 20 mg/l (18). The PCT-based algorithm was considered positive when antibiotics treatment was indicated only.

### Laboratory Tests

Blood samples were obtained from umbilical cord blood in standard vacuum blood collection tubes (TERUMO) containing lithium heparin. The tubes were transported to the biochemistry laboratory within 1 h; then, PCT level was assessed with the KRYPTOR automated analyzer (BRAHMS, Hennigsdorf, Germany), and CRP level was assessed with the COBAS 6000 analyzer (Roche Diagnostics, Mannheim, Germany).

### Data Collection and Statistical Analysis

Baseline characteristics of all newborns with suspected EONI are described with frequencies (%) and 95% confidence intervals (CIs) for categorical data and medians (interquartile range) or means (SD) for quantitative data. Sensitivity, specificity, and negative and positive predictive values and negative and positive likelihood ratios were estimated for both algorithms. A newborn was considered infected when a proven or a probable infection was confirmed. An algorithm was considered “positive” when antibiotics treatment was indicated. Diagnostic performance was considered significantly different when 95% CIs did not overlap. These diagnostic values were compared by the χ^2^ test with a significance limit of *p* < 0.05. Statistical analyses were made with the Stata Statistical software version 11 (StataCorp. 2009).

## Results

**Figure 2:**
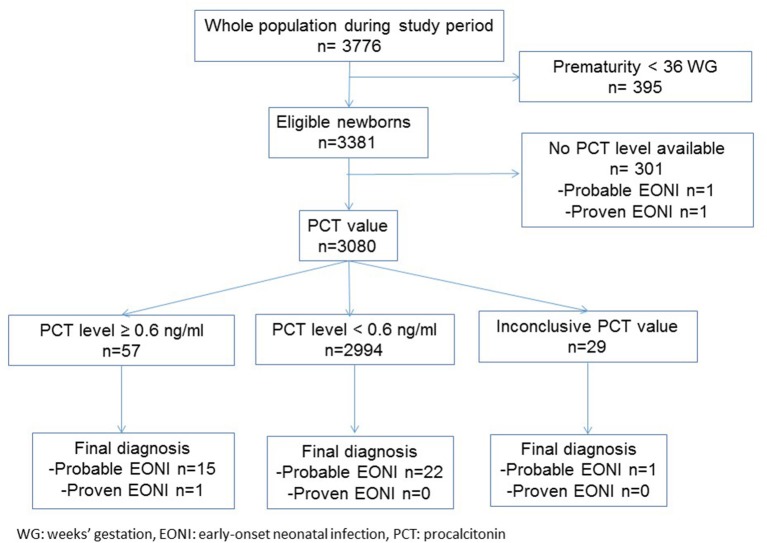
Flowchart of newborns in the study.

We included 3,080 of the 18,542 births that occurred during the study period from March 1, 2011 to September 30, 2012 in the three French birth centers (Figure 2). Only 1 newborn had certain infection, and 37 newborns had probable infection. The global exposure to antibiotics treatment was 142/3,080 (4.6%; 95% CI, 4.1–5.0) with a significantly different distribution among the centers, from 3.8% (59/1,556; 95% CI, 2.8–4.7) in Center A to 6.2% (22/357; 95% CI, 3.7–8.7) in Center B (*p* < 0.05). Among the 1,571 (51%) women with intrapartum antibiotic treatment, 689 (44%) received 2 doses, whereas 882 (56%) received one dose only.

The newborn with certain infection had a positive GBS blood culture. For the 37 newborns with possible infection, GBS was found in 10 gastric fluids and 4 placental cultures. *Escherichia coli* was found in three gastric fluids and two placental cultures. The characteristics of included newborns are in (Table 1). The population differed among the centers regarding gestational age, infectious risk factors, and clinical symptoms. The excluded population is described in (Supplementary Table 1).

**Table 1:** Characteristics of the study population by center, biomarkers values, and antibiotic rates.

	**Center A (*n* = 1,556)**	**Center B (*n* = 357)**	**Center C (*n* = 1,167)**	**Total (*n* = 3,080)**	***P*-value**
Chorioamnionitis	1 (0.06)	8 (2.2)	1 (0.08)	10 (0.3)	**<10–**^**4**^
Maternal fever before or at the beginning of labour > 38°C	76 (5.0)	24 (6.7)	83 (7.1)	183 (5.9)	** <10–**^**4**^
Time of membrane rupture					
>18 h	253 (16.2)	65 (18.2)	271 (23.2)	589 (19.1)	**<10–**^**4**^
12 to 18 h	146 (9.4)	40 (11.2)	335 (28.7)	521 (16.9)	**<10–**^**4**^
PROM before 37 WG without maternal antibiotic treatment	13 (0.8)	34 (9.5)	60 (5.1)	107 (3.5)	**<10–**^**4**^
Abnormalities of the foetal heart rate or unexplained perinatal anoxia	296 (19.0)	41 (11.5)	412 (35.3)	749 (24.3)	**<10–**^**4**^
Meconial or stained amniotic fluid without obstetrical cause	490 (31.5)	155 (43.4)	310 (26.6)	955 (31.0)	**<10–**^**4**^
Prematurity <37 or ≥35 WG	46 (2.9)	8 (2.2)	50 (4.3)	104 (3.4)	**0.002**
Newborn fever	1 (0.06)	11 (3.1)	0	12 (0.4)	**0.02**
Symptomatic	124 (7.9)	20 (5.6)	80 (6.8)	224 (7.3)	0.36
Hemodynamic symptoms	19 (1.2)	1 (0.3)	2 (0.2)	22 (0.7)	**5.10**^**4**^
Respiratory symptoms	2 (0.1)	6 (1.7)	4 (0.3)	12 (0.4)	0.42
GBS-positive vaginal swab without maternal treatment	87 (5.6)	22 (6.2)	31 (2.6)	140 (4.5)	**0.002**
GBS-positive vaginal swab with maternal treatment	346 (22.2)	96 (27.0)	64 (5.5)	506 (16.4)	**<10–**^**4**^
No vaginal swab results	121 (7.8)	35 (1.0)	190 (16.3)	346 (11.2)	**<10–**^**4**^
CRP level, mean (SD)	7.6 (12.6)	3.7 (6.6)	8.7 (13.8)	6.4 (11.3)	
Median (IQR)	3 (3–4)	2 (2–3.3)	5 (5–6.1)	3 (2.3–5)	
PCT level, mean (SD)	0.2 (1.7)	0.4 (3)	0.2 (1)	0.2(1.5)	
Median (IQR)	0.13 (0.11–0.16)	0.13 (0.11–0.18)	0.14 (0.12–0.17)	0.14 (0.11–0.17)	
Neonatal antibiotic rate	59 (3.8)	22 (6.2)	61 (5.2)	142 (4.6)	
Absolute change in neonatal antibiotics prescription rate by using the PCT algorithm vs. current practice	2.1%	1.2%	1.5%	1.8%	
Relative change in neonatal antibiotics prescription rate by using the PCT algorithm vs. current practice	55.2%	19.3%	28.8%	39%	

*Categorical variables are expressed with number (%) and continuous variables with mean (SD) or median (interquartile range [IQR])*.

*GBS, group B streptococcus, PROM, premature rupture of membranes; WG, weeks' gestation; CRP, C-reactive protein; PCT, procalcitonin*.

### Cord-Blood PCT Value

Among 3,051 available PCT values, 57 were positive (PCT level ≥ 0.6 ng/ml), but only one newborn had proven EONI and 15 newborns had probable infection (Figure 2). Among 2,994 newborns with PCT level <0.6 ng/ml, none had proven infection and 21 had probable infection. For probable neonatal bacterial infection, the sensitivity and specificity of the cord-blood PCT value were 42% (95% CI, 40.3–43.8) and 98.7% (95% CI, 98.3–99.1), respectively. The positive and negative predictive values were 26.3 and 99.2%, respectively. The positive and negative likelihood ratios were 31 (95% CI, 19–50) and 0.6 (95% CI, 0.45–0.77), and positive and negative posttest probabilities were 28 (95% CI, 19–39) and 1 (95% CI, 1–1).

Most of the probably infected newborns had a healthy clinical presentation but were considered as probably infected because they had (i) transitory symptoms at birth but quickly resolving 2 h later, (ii) a positive Gram coloration analysis of their gastric fluid (no more recommended in France since October 2017), or (iii) the association with a mild to moderate inflammatory biomarkers elevation. The specific clinical and biological descriptions of this population are now detailed in (Table 2). The only newborn with proven infection was born asymptomatic at 41 weeks' gestation with a positive PCT value (PCT level, 0.91 ng/l) and CRP level of 9.7 mg/L at 12 h after birth. Prenatal vaginal screening for GBS was not performed, and the labor was spontaneous, with vaginal delivery after one dose of intrapartum antibiotics only. The Apgar was 10/10/10, but at 4 h after birth, desaturation was noticed and amoxicillin and amikacin antibiotics were initiated. A blood culture assessed at 4 h after birth was positive for GBS (positivity in 10 h), and the placental culture was also positive for GBS.

**Table 2:** Clinical, biological, and bacteriological data of probable infected newborns with negative PCT level (<0.6 ng/ml).

**Newborns**	**Gestational age (GA)**	**Birth weight (g)**	**GBS vaginal swab**	**Intra partum antibiotic**	**Apgar score at 5 min**	**Persistent or new clinical symptoms after H2**	**Gastric fluid culture**	**Blood culture**	**CRP value (mg/l)**	**PCT value (ng/ml)**	**Outcome**
Clinical symptoms at birth	37	3,500	No	Yes	10	No	E.Coli	Negative	3	0.13	Moderate sequalae
	38	3,760	Yes	Yes	10	No	Negative	Negative	16	0.14	Healthy
	39	3,310	No	No	10	No	Negative	Negative	24	0.14	Healthy
	39	3,320	Yes	No	10	No	Streptococcus B	Negative	33	0.12	Healthy
	40	3,270	No	No	10	No	Negative	Negative	20	0.15	Healthy
	40	3,535	No	Yes	10	No	Streptococcus B	Negative	55	0.18	Healthy
	40	3,340	Yes	Yes	10	No	Negative	Negative	66	0.1	Healthy
	41	3,220	No	No	8	No	Negative	Negative	2	0.11	Healthy
	41	3,450	No	No	10	No	Negative	Negative	NC	0.16	Healthy
Asymptomatic at birth	38	2,870	Yes	Yes	9	No	Negative	Micrococcus Luteus	11	0.1	Healthy
	39	3,010	Yes	Yes	10	Hemodynamic	Negative	Negative	68	0.1	Healthy
	39	2,700	No	Yes	10	No	Negative	Negative	34.7	0.19	Healthy
	39	2,735	No	No	10	No	Negative	Not done	NC	0.11	Healthy
	39	3,725	No	Yes	10	No	Negative	Negative	31	0.19	Healthy
	40	3,400	Yes	Yes	10	No	Streptococcus B	Negative	11	0.16	Healthy
	40	3,745	No	No	10	No	Streptococcus B	Not done	31	0.31	Healthy
	40	3,720	Yes	Yes	10	Hemodynamic	Negative	Negative	27	0.12	Healthy
	40	3,325	No	No	10	No	Streptococcus B	Negative	17.8	0.24	Healthy
	41	3,640	No	No	8	No	Negative	Negative	10	0.1	Healthy
	41	3,800	No	No	10	No	Negative	Not done	12	0.16	Healthy
	41	3,660	No	No	9	No	Negative	Negative	30	0.32	Healthy
	41	3,710	No	No	10	No	Streptococcus sanguinis	Negative	3	0.55	Healthy

### Diagnostic Value of PCT-Based Algorithm

Among the 224 symptomatic newborns, only 87 presented serious symptoms or persisting symptoms beyond 2 h after birth. Among the 137 asymptomatic newborns, only 2 had PCT level ≥ 0.6 ng/L and only one CRP level > 20 mg/L. According to the PCT-based algorithm, among the 88/3,080 patients with an indication for antibiotics prescription, 17 had probable infection and 1 proven infection. The sensitivity and specificity of the PCT-based algorithm were 48% (95% CI, 46.2–49.8) and 97.7% (95% CI, 97.1–98.2), respectively. The positive and negative likelihood ratios were 21 (95% CI, 14–28) and 0.53 (95% CI, 0–1.9), respectively, and positive and negative posttest probabilities were 20 (95%, CI 15–28) and 1 (95% CI, 0–1), respectively.

### Antibiotics Exposure with the PCT-Based Algorithm

According to the PCT-based algorithm, the proportion of antibiotics prescriptions would be 2.8% (88/3,080; 95% CI, 2.4–3.1), corresponding to a 1.8% (95% CI, 1.3–2.3) potential global decrease in neonatal antibiotics prescription as compared with the proportion in terms of current practice in these newborns, 4.6% (142/3,080; 95% CI, 4.1–5.0). The relative decrease in prescriptions with the PCT-based algorithm would be 39% (95% CI, 37.3–40.7).

The absolute and relative changes in neonatal antibiotics prescription rate with the PCT-based algorithm vs. current practice are in (Table 1). According to the new national guidelines that recommend treatment for all symptomatic newborns, the proportion of indications for antibiotics prescriptions would be 7.2% (224/3,080; 95% CI, 6.7–7.7) in this selected population of newborns with EONI risk factors.

## Discussion

Our validation study of the PCT-based algorithm confirms a potential decrease in neonatal antibiotics exposure with the algorithm (from 4.6 to 2.8%, or 1.8% decrease). The current practice of antibiotics prescription that we found is similar to national and international practices in this population of newborns (5). The new national and international guidelines for managing suspected EONI in newborns encourage limiting antibiotics to only symptomatic newborns (21), but our results suggest that an umbilical-cord-blood PCT value could further help in limiting antibiotics prescriptions in a personalized risk stratification approach. However, this study is not designed to demonstrate non-inferiority on neonatal morbidity or mortality. This demonstration has become particularly difficult because of the very low incidence of EONI (0.5/1,000 births) and would require a very large multicentric study including thousands of newborns (3).

We observed some degree of lower diagnostic value of the cord-blood PCT assay, with 40% sensitivity and 98.7% specificity, as compared with Joram et al. in a monocentric study of 2,151 newborns with suspected EONI, the sensitivity and specificity of the algorithm were 92% (95% CI, 0.75–0.98) and 97% (95% CI, 0.96–0.98), respectively. This result underlines the importance of external validation studies to determine the diagnostic value of a test and its utility and potential impact in clinical practice. Recently, a meta-analysis including 17 reviews and 2,197 episodes of suspected neonatal infection [78 documented as systemic infection (17.2%) (22)] reported the sensitivity and specificity of diagnosis with a cord-blood PCT value of 82% (95% CI, 0.72–0.89) and 86% (95% CI, 0.58–0.96). The high rate of probable EONI cases as compared with the only certain EONI case in our study population could explain the difference in sensitivity (possible variation in the gold-standard definition resulting in an increased false-negative rate). Altogether, these results argue for integrating the umbilical-cord-blood PCT value in a decision algorithm or clinical decision rule rather than for isolated interpretation and use.

### Weaknesses and Strengths

Even in this large multicentric study, the very low prevalence of the proven EONI case (1/3,080 = 0.32/1,000 births in a selected risk population) is responsible for a lack of power and subsequent lack of precision in diagnostic value measurement with large 95% CI. However, this prevalence is consistent with the previously reported values of EONI (8). Moreover, 301/3,381 (8.9%) newborns were excluded from this study because the cord-blood PCT value was not assessed (Supplementary Table 1). The significant differences observed between these newborns and those with an available PCT value suggest a more frequent context of emergency during the delivery (chorioamnionitis, maternal fever, abnormalities of fetal heart rate, meconial amniotic fluid) and an increased rate of symptomatic newborns (fever, respiratory distress) and a possible feasibility limit because of emergency and busy activity. Regardless, this situation should not introduce a serious bias because these symptomatic children require systematic antibiotics therapy. Moreover, the diagnostic value of PCT-based algorithm in this study is lower than that already published by Lencot et al. when the algorithm was initially derived as the result of a high number of “misclassification” among the probably infected newborns (7). However, the main objective of such a diagnostic approach is not to definitively rule out a neonatal early bacterial infection (all the newborns at risk of infection are under close surveillance in the maternity ward during the first 72 postnatal hours) but rather to improve the stratification of the infectious risk in this specific newborns population exposed to current overdiagnosis and subsequent overtreatment. In addition, we hypothesized that most of the probably infected newborns in this study were not really “missed” by the PCT-based algorithm but possibly not all were really infected, underlying the problem of the lack of a robust reference test in this pathology.

Center C already had experience in using the PCT assay in clinical practice for a long time, but the interpretation of the value was not integrated into an algorithm and the antibiotics prescription was left to the discretion of the clinicians. This scenario could have reduced the baseline antibiotics prescription rate: 5.2% (61/1,167) of newborns in this center were exposed to antibiotics as compared with 3.7% (43/1,167) in Center A. Of note, the three experts who classified EONI *a posteriori* in our study were blinded to PCT results.

The antibiotics exposure significantly differed among the centers: 3.8% (59/1,556; 95% CI, 2.8–4.7) for Center A, 6.2% (22/357; 95% CI, 3.7–8.7) for Center B, and 5.2% (61/1,667; 95% CI, 3.9–6.5) for Center C. This finding underlines the difficulty in implementing national guidelines but can also be explained by the fact that the prematurity rate in the study population differed among the centers (2.9% in Center A and 4.3% in Center C), which resulted in more symptomatic newborns and more antibiotics indications.

### Implications for Practice

The new PCT-based algorithm proposed by S. Lencot would lead to a potential 1.8% (95% CI, 1.3–2.3) absolute decrease and 39% (95% CI, 37.3–40.7) relative decrease in neonatal antibiotics exposure not only as compared with current practice but also as compared with the new French guidelines. This control in antibiotics use could limit the deleterious effects on children and future adult health specifically by limiting the perturbations in newborn microbiota and the selection of antibiotic-resistant bacteria (8, 10, 11). If confirmed in an ongoing impact comparative study, the umbilical-cord-blood PCT value could be a useful tool in evaluating personalized risk stratification to limit neonatal antibiotics exposure.

## Data Availability Statement

The raw data supporting the conclusions of this article will be made available by the authors, without undue reservation, to any qualified researcher.

## Ethics Statement

The studies involving human participants were reviewed and approved by the local clinical research committee of Nantes University Hospital. Written informed consent to participate in this study was provided by the participants' legal guardian/next of kin. Written informed consent was not obtained from the minor(s)' legal guardian/next of kin for the publication of any potentially identifiable images or data included in this article.

## Author Contributions

Research director: CG. Writing and data collection: NH. Data collection rereading: EL, GG, BL, CS, JM, DM, SD, CB, and JC.

## Conflict of Interest

The authors declare that the research was conducted in the absence of any commercial or financial relationships that could be construed as a potential conflict of interest.
