# Supplementary material for: Potential Impact of Umbilical-Cord-Blood Procalcitonin-Based Algorithm on Antibiotics Exposure in Neonates With Suspected Early-Onset Sepsis
Source: Front Pediatr. 2020 Apr 17;8:127. doi: 10.3389/fped.2020.00127 (PMC7181674; doi:10.3389/fped.2020.00127)
Supplement: Supplementary file 1 [file Table_1.docx]

Supplemental table 1: Characteristics of included and excluded eligible newborns (no PCT value available)

|  | Included  (n=3080) | Excluded  (n=301) | P value |
| --- | --- | --- | --- |
| Chorioamnionitis | 10 (0.3) | 11 (3.6) | **0.002** |
| Maternal fever before or at the beginning of labour > 38°C | 183 (5.9) | 17 (5.6) | **0.004** |
| Time of membrane rupture |  |  |  |
| > 18 hr | 589 (19.1) | 51 (17.0) | 0.08 |
| 12 to 18 hr | 521 (16.9) | 30 (10.0) | **0.005** |
| PROM before 37 WG without maternal antibiotic treatment | 107 (3.5) | 60 (19.9) | **<10^-4^** |
| Abnormalities of the foetal heart rate or unexplained perinatal anoxia | 749 (24.3) | 88 (29.2) | **0.03** |
| Meconial or stained amniotic fluid without obstetrical cause | 955 (31.0) | 135 (44.8) | **<10^-4^** |
| Prematurity < 37 or ≥ 35 WG | 104 (3.4) | 14 (4.6) | 0.1 |
| Newborn fever | 12 (0.4) | 16 (5.3) | **<10^-4^** |
| Hemodynamic symptom | 22 (0.7) | 1 (0.3) | 0.37 |
| Respiratory symptom | 12 (0.4) | 8 (2.6) | **<10^-4^** |
| GBS-positive vaginal swab without maternal treatment | 140 (4.5) | 7 (2.3) | **<10^-4^** |
| No vaginal swab results | 346 (11.2) | 28 (9.3) | **0.02** |
| CRP level, mean (SD)  Median (IQR) | 6.4 (11.3)  3 (2.3-5) | 3.5 (3.87)  2 (2-3.3) |  |
| PCT level, mean (SD)  Median (IQR) | 0.2 (1.5)  0.14 (0.11-0.17) | 0 |  |

GBS: group B streptococcus, PROM: premature rupture of membranes, WG: weeks’ gestation; CRP: C-reactive protein; PCT: procalcitonin

Categorical variables are expressed with number (%) and continuous variables with mean (SD) or median (interquartile range [IQR])
